# Mendelian randomization study reveals the causal effect of sex hormone binding globulin on osteoporosis

**DOI:** 10.1186/s12891-025-08956-7

**Published:** 2025-07-26

**Authors:** Guo-Si-Lang Zuo, Yu Huang, Ze Wang, Hong Wang, Fashuai Wu

**Affiliations:** 1 Department of Orthopaedics, Union Hospital, Tongji Medical College, Huazhong University of Science and Technology, Wuhan, 430022 China; 2 Department of Otorhinolaryngology, The Third Hospital of Wuhan City, Wuhan, 430070 China

**Keywords:** Two-sample Mendelian randomization, Sex hormone binding globulin, Bone mineral density, Osteoporosis

## Abstract

Multiple observational studies have reported the relationship between circulating SHBG level and bone health. However, their results were inconclusive, in this research we aim to exam the relationship by two-sample Mendelian randomization. Several quality control steps were taken in our analysis to select eligible instrumental SNPs strongly associated with circulating SHBG level. Our analysis employed several robust analytical methods (inverse-variance weighting, weighted median, MR-Egger regression and MR.RAPS method) to enhance the reliability of causal inferences. To assess the horizontal pleiotropy, heterogeneities and stability of these genetic variants on BMD, MR-Egger intercept test, Cochran’s Q test and “leave-one-out” sensitivity analysis were performed during analysis. To reduce heterogeneity and the effect of horizontal pleiotropy, outlier variants identified by MR-PRESSO outlier test were excluded. Multivariable MR analysis was performed to control for potential horizontal pleiotropy acting via BMI or T2DM. Reverse MR analysis was performed to guard against the possibility of reverse causality. Our two-sample Mendelian randomization analyses with two groups of exposure (circulating SHBG) GWAS summary statistics and four groups of outcome (BMD of different skeletal sites) GWAS summary statistics suggests an inverse link between circulating SHBG level and BMDs of different skeletal sites. Results of multivariable MR analysis demonstrates that the inverse link between circulating SHBG level and BMDs is independent of BMI and T2DM. Reverse MR analyses demonstrates no reverse link between TB-BMD and circulating SHBG level. Our result can be helpful for researchers to identify new treatment target for osteoporosis, or reminding clinicians of taking measures and concerted efforts to prevent or intervene with bone loss when patients are diagnosed as high circulating SHBG level.

**Supplementary Information:**

The online version contains supplementary material available at 10.1186/s12891-025-08956-7.

## Introduction

Osteoporosis, a prevalent clinical skeletal disorder mainly affecting postmenopausal women and elderly male patients is clinically diagnosed via bone mineral density (BMD) measurement at central skeletal sites (the lumbar spine and the proximal femur) and peripheral skeletal sites (including the distal forearm) using dual-energy X-ray absorptiometry (DXA) [1, 2]. Characterized by reduced BMD and structural deterioration of bone tissue, osteoporosis is closely associated with fracture, especially fracture of the hip, spine, and wrist [3, 4]. Therefore, osteoporosis may lead to diminished quality of life, increased economic burden, and elevated disability and mortality [5]. Clearly, a concerted effort is urgently needed to identify risk factors for osteoporosis and to assess patients at risk of fracture to allow for prevention and early intervention when appropriate. It is commonly acknowledged that aging, hormonal imbalance, low body mass index (BMI), physical inactivity, smoking, heavy alcohol consumption, inflammatory bowel disease, diabetes mellitus, calcium and vitamin D deficiency etc. and many genetic factors are correlated with osteoporosis [1, 6]. Substantial observational evidences demonstrates that endogenous sex steroid hormones (not only serum testosterone (T) and estradiol (E2) but also their transporter - sex hormone-binding globulin (SHBG)) are closely associated with the occurrence and development of osteoporosis [7–10]. Factors conferring elevated lifetime circulating sex steroid hormones concentrations, such as early age at menarche, long reproductive life, late age at menopause and hormone replacement therapy, have been recognized as well-established protective factors against osteoporosis [11, 12]. Conversely, diminished sex steroid levels result in reduced BMD and elevated risk of fracture [13].

SHBG is a homodimeric plasma glycoprotein produced by hepatocytes. It functions through binding with high affinity and selectivity for estrogens and/or androgens in the blood, and thus it is regarded as a transporter or reservoir for sex steroids [14].The concentration of SHBG in human blood critically regulates the amounts of free (bioactive) androgens and estrogens available to passively diffuse into tissues and access to their target cells [15, 16]. Over the past two decades, research has revealed that SHBG binds to specific receptors expressed on the cell membranes of various tissues and is also present intracellularly [17]. Beyond its classical role of SHBG on modulating passive diffusion of sex hormone, SHBG binding to its receptor activates cyclic adenosine monophosphate (cAMP) signaling, a pathway implicated in promoting BMD [17, 18]. Thus, SHBG perhaps possesses pleiotropic actions in bone health. A cross-sectional study among 404 Chinese middle-aged and elderly men reported that high SHBG was an independent risk factor of osteoporosis/osteopenia after adjustment for E2 and T [10]. High SHBG concentration was recognized as a risk factor of bone loss and fracture in postmenopausal women according to a study by Bjørnerem et al. [19]. While some reports showed positive or no significant relation between SHBG level and BMD [20–22]. A study of 927 women and 894 men followed up for 6.5 years showed that serum SHBG level was significantly correlated with bone loss assessed by wrist BMD, but this correlation disappeared in women after adjustment for BMI [23]. Araujo’s study found SHBG was inversely correlated with total hip and ultra distal radius BMD after adjusting for age, but not with multivariate adjustment for the factors of age, lean mass, fat mass, physical activity, self-rated health, and smoking [24]. An animal study reported that the high SHBG genotype was positively associated with BMD value, furthermore, the male mice over-expressing SHBG had increased femoral cortical bone mineral content [25].

The aforementioned observational studies, which reached inconsistent conclusions, are either based on limited samples or only explore the correlation between SHBG level and osteoporosis. Residual confounding, reverse causation and exposure measurement error occur frequently in these studies, which potentially bias the results and hinder us from making solid and robust causal inference [26]. Thus, study like randomized controlled trial (RCT) is needed to establish a definitive causal link between SHBG level and osteoporosis. However, RCT is difficult or impractical to perform owing to its labor resource-intensive and time-consuming nature, and crucially, the ethical constraints involved in experimentally manipulating circulating SHBG levels in experimental human group. Mendelian randomization (MR) study offers a powerful and convenient alternative approach to both conventional observational studies and infeasible RCT studies. MR utilizes genetic variants as instrumental variables to test the causal effect of an exposure (e.g., circulating SHBG level) on an outcome (e.g., BMD or osteoporosis risk) [26]. This approach holds significant promise for elucidating the etiology of osteoporosis and identifying potential strategies for preventing and intervening with bone loss.

Mendelian randomization analysis mimics the design of RCT, using genetic variants that robustly associated with the exposure of interest in an instrumental variable analysis to test the causal relationship between the exposure and outcome [27]. This approach harnesses publicly accessible large genome-wide association studies (GWAS) data for both risk factor “exposure” and disease “outcome” and it overcomes the typical pitfalls present in conventional observational studies and RCT studies. To obtain unbiased estimates, MR studies must satisfy three key assumptions: (1) the genetic variants used as instrumental variables must exhibit robust associations with the exposure; (2) these variants extracted as instrument variables for exposure must be independent of confounders affecting both the exposure and the outcome; and (3) the variants must influence the outcome exclusively through the exposure, with no horizontal pleiotropy (i.e., no effect via alternative biological pathways) [28].

The aim of our study was to clarify the causal link between genetically determined circulating SHBG level and BMDs of different skeletal sites under the two-sample MR analysis framework, in which we used the publicly available GWAS data for SHBG and BMD. Besides, as some of the selected genetic variants for SHBG are associated with BMI or T2DM, we performed multivariable MR analysis to control for potential horizontal pleiotropy mediated by these traits [29]. Additionally, reverse MR analysis also was performed to guard against the possibility that our results were driven by the reverse causality [30, 31].

## Materials and methods

### Selection of genetic variants associated with Circulating SHBG

We selected 16 single-nucleotide polymorphisms (SNPs) significantly associated with circulating SHBG level as the first set of instrument variables to investigate the causal relationships between circulating SHBG level and specific outcome in the research. The process of identifying IVs of circulating SHBG involved three steps. At first step, initial identification: publications reporting SNPs associated with circulating SHBG level at genome-wide significance (*P* < 5 × 10^−8^) were searched, and the SNPs were retrieved [32, 33]. Secondly, expansion of IVs: to increase the number of instrument variables, and thereby reduce potential weak instrument bias, the GWAS catalog (https://www.ebi.ac.uk/gwas/home) was additionally searched to retrieve more eligible instrumental SNPs related to circulating SHBG level at a relaxed threshold (P < = 6 × 10^−6^). At last, summary data acquisition: we acquired summary data (i.e., allele frequency, beta value, standard error, and P values) of the 16 SNPs identified at first step from a large GWAS dataset comprising 28,837 individuals (13,899 women and 14,938 men) of European ancestry [34]. The characteristics of the selected 16 SNPs for circulating SHBG level was presented in Additional File 1. Estimates for these IVs had been adjusted for age, sex and BMI. For replication purposes, a recent larger GWAS of circulating SHBG level involving 312,215 European participants (males and females) also was incorporated in our analysis. Full summary statistics for this larger GWAS are publicly available and can be downloaded from the UK biobank (http://www.nealelab.is/blog/2019/9/16/biomarkers-gwas-results) and IEU GWAS database (https://gwas.mrcieu.ac.uk/). Estimates of SNPs in the GWAS had been adjusted for many common-seen components.

### BMD GWAS summary statistics

To investigate the causal relationship between circulating SHBG level and bone mineral density, several publicly available GWAS summary statistics for BMD were downloaded from the GEnetic Factors for OSteoporosis Consortium (GEFOS: http://www.gefos.org/). Estimates for SNPs in these BMD GWAS datasets had been adjusted for several common-seen components, such as: sex, age, height and weight etc.

Specifically, summary statistics from three separate European-ancestry GWAS datasets were downloaded from GEFOS and utilized in our analysis, which include Femoral Neck bone mineral density (FN-BMD, *n* = 32735), Lumbar Spine bone mineral density (LS-BMD, *n* = 28498), and Forearm bone mineral density (FA-BMD, *n* = 8143). These represented the largest available GWAS statistics for DXA-measured BMD at the time of our analysis [6].

Additionally, the GWAS summary statistics for Total Body-bone mineral density (TB-BMD) derived from a meta-analysis of 56,284 individuals of European ancestry was downloaded from the GEFOS website, and the meta-analyzed effect size estimates were used in our study [35].

### BMI and T2DM GWAS summary statistics

We employed a multivariable inverse-variance weighted (IVW) MR approach to adjust for potential horizontal pleiotropy acting through BMI and T2DM. Results of multivariable MR analysis also could demonstrate whether BMI or T2DM may mediate the causal effect of circulating SHBG level on osteoporosis or not [36, 37]. Genetic data of 322,154 European descents for BMI was publicly available and was retrieved from the GIANT consortium [38]. For T2DM, we extracted its summary data from a reanalysis dataset, which included 70,127 (12,931 cases and 57,196 controls) individuals of European descent [39]. The association results were available at the Type 2 Diabetes Knowledge portal (www.type2diabetesgenetics.org/) and the complete summary statistics was publicly available and could be downloaded at http://cg.bsc.es/70kfort2d/.

### Validation of genetic instrumental variables

In this two-sample MR study, we ensured independence of instrumental variables by performing the clumping process, which could estimate LD (linkage disequilibrium) between instrumental SNPs, using the European samples from the 1000 genomes project. This step is essential as instrumental SNPs for the exposure in strong linkage disequilibrium (LD) can bias results. Pairwise LD between these SNPs would be calculated. With the window size of 10,000 kb, for any SNP pair with an LD R² >0.001, the SNP exhibiting the stronger association with the exposure (smaller p-value) would be retained for next step. These steps yielded a set of instrumental SNPs largely independent within the specified LD threshold.

To minimize potential violations of the key MR assumption that instrumental SNPs influence the outcome solely through the exposure and not via other biological pathways (i.e., absence of horizontal pleiotropy), SNPs significantly associated with the outcome disease after Bonferroni correction (*p* < 0.05/N, where N is the total number of SNPs queried) were excluded [40]. By default, if a particular requested SNP was not searched in the outcome GWAS, a proxy SNP in LD (r² >0.8, based on 1000 genomes European sample data) with the requested SNP was identified and used instead. For such proxies, the outcome GWAS association statistics (effect size, standard error, p-value) for the proxy SNP were returned. Crucially, along with the proxy SNP, the effect allele of the proxy SNP and its corresponding allele for the target SNP (in phase) were also returned to ensure accurate effect alignment. To ensure the effect of a SNP on the exposure, and the effect of that same SNP on the outcome, corresponded to the same allele, the effect of ambiguous SNPs with non-concordant alleles (e.g., A/G vs. A/C) and palindromic SNPs with ambiguous strand (e.g., A/T or G/C) would be corrected or the ambiguous and palindromic SNPs would be directly excluded from the selected instrument SNPs mentioned above during the harmonizing process. These resulting set of stringently selected and harmonized SNPs served as instrumental variables for the subsequent two-sample MR analysis. To assess potential bias from weak instrumental variables (i.e., genetic variants selected as instrumental variables with weak associations with the exposure), we calculated the F statistic using the formula (F = R^2^(n-k-1)/k(1-R^2^), R^2^: variance of exposure explained by selected instrumental variables, which was obtained from MR Steiger directionality test, n: sample size, k: number of instrument variables) [41, 42]. An F statistic substantially greater than 10 indicates a low risk of weak instrument bias [43].

### Mendelian randomization analyses

Mendelian Randomization employs genetic variants as instrumental variables to estimate the causal effect of an exposure (circulating SHBG levels) on outcomes (BMDs). We combined summary statistics (β coefficients and standard errors) from genome-wide association studies (GWAS) for this purpose. Given the potential for invalid instruments due to pleiotropy, we employed several robust MR methods, each relying on distinct assumptions. The methods included inverse variance weighting (IVW), MR-Pleiotropy RESidual Sum and Outlier (MR-PRESSO) method, weighted median (WMM), MR-Egger regression and Robust Adjusted Profile Score (MR.RAPS). The IVW method combines wald ratio estimates for each SNP (i.e., the β coefficient of the SNP for BMD divided by the β coefficient of the SNP for circulating SHBG) using meta-analysis approach, yielding an overall causal estimate of the effect of circulating SHBG on BMD [44]. This method provides an unbiased causal estimate under the assumption of no horizontal pleiotropy or balanced pleiotropy [45]. We used a random-effects IVW model if significant heterogeneity was detected (*P* < 0.05), otherwise, a fixed-effects model was applied. MR-PRESSO performs (a) a global test for horizontal pleiotropy; (b) outlier test for correcting horizontal pleiotropy via outlier removal; and (c) a distortion test testing of significant differences in the causal estimates before and after outliers correction. The MR-PRESSO outlier test relied on the InSIDE (Instrument Strength Independent of Direct Effect) condition that instrument-exposure and pleiotropic effects were uncorrelated, and it required balanced pleiotropy, with at least 50% valid instrument variants [46]. The median based approach would provide an unbiased causal estimate even when up to 50% of SNPs were invalid instrument variables due to unbalanced horizontal pleiotropy [47]. MR-Egger regression, possessing the strength of reducing bias introduced by a particular direction of the horizontal pleiotropic effect, allows for a non-zero intercept, and provides a valid test of the null causal hypothesis and a consistent causal effect estimate under the InSIDE assumption, even if all instruments are invalid instrument variables [48]. While MR-Egger regression provides valuable correction for directional pleiotropy, its estimates can be sensitive to outliers. Compared with MR-Egger estimates, the WMM estimate which did not require the InSIDE assumption has been shown to have distinct superiorities for its improved power of detecting causal effect and maintaining lower type I error [47]. Correcting for pleiotropy using robust adjusted profile scores, MR.RAPS is robust to both systematic and idiosyncratic pleiotropy and performs well for MR analysis even with many weak instruments [49]. Besides, it was highly recommended to routinely use MR.RAPS method in MR analysis to enhance result robustness, especially when the exposure and the outcome were both complex traits and many instrumental variables were used. Each method described above possessed its own strengths and shortcomings, thus we combined the different methods in the analysis. Significance of the causal association was established under two scenarios. if there was no evidence of directional pleiotropy (P for MR-Egger intercept > 0.05) and the estimates of different methods were inconsistent, an association between exposure and outcome phenotype was considered significant with its adjusted *P*-value < 0.0125 (Bonferroni-corrected threshold for 4 tests). If there was an evidence of directional pleiotropy presence (P for MR-Egger intercept < 0.05), the MR-RAPS estimate was deemed reliable, given its robustness to both systematic and idiosyncratic pleiotropy.

### Pleiotropy, heterogeneity and sensitivity analysis

Horizontal pleiotropy was assessed using MR-Egger regression, where the intercept term served as an indicator of directional pleiotropy whether driving the results of an MR analysis or not [50]. Heterogeneity was evaluated using Cochran’s Q statistic, with *P* < 0.05 considered statistically significant heterogeneity. To address heterogeneity, MR-PRESSO analysis was performed with the number of distributions was set to 1,000, systematically removing outlier SNPs that disproportionately contributed to heterogeneity. Additionally, leave-one-out sensitivity analysis was conducted to identify potentially influential SNPs driving the causal link between exposure and outcome, by iteratively excluding individual variants and re-running the MR analysis.

### Procedures of MR analysis

**Fig. 1:**
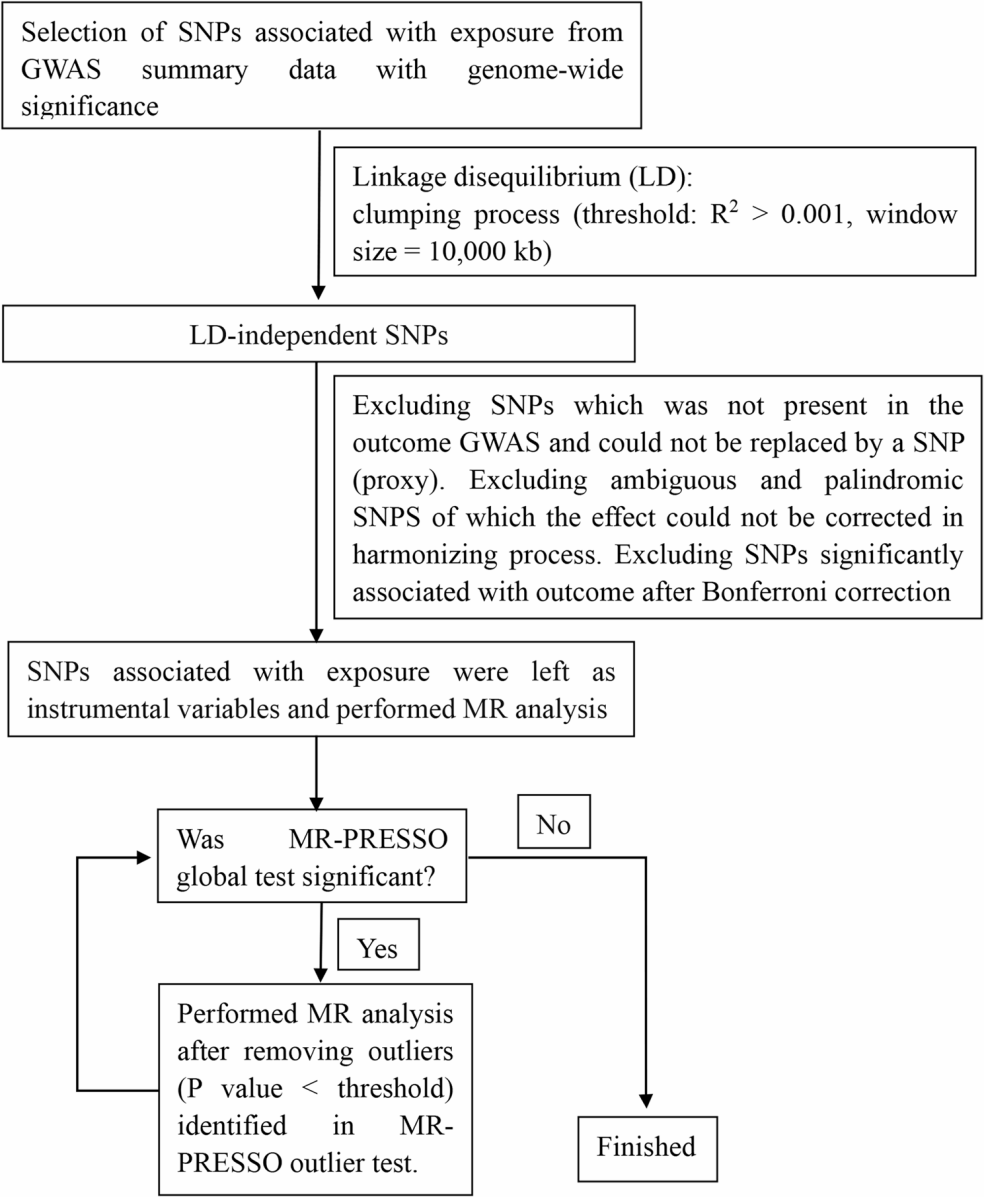
A flow chart about the analytical methods and the procedures of MR analysis

SNPs significantly associated with the outcome (after Bonferroni correction) were excluded prior to analysis. MR analysis was initially performed using the remaining SNPs. Where MR-PRESSO identified significant horizontal pleiotropy, outlier variants were excluded, and MR analysis was repeated to minimize heterogeneity. A flowchart of the analytical procedure is presented in Fig. 1. STROBE-MR checklist of recommended items was adopted to guide our MR analysis [51].

### Causal directional test and multivariable MR analysis

Reverse MR analysis was conducted to assess whether genetically predicted TB-BMD causally influences circulating SHBG level, thereby testing for reverse causation. SNPs with a marginal *p* value smaller than 5 × 10^−8^ were selected as instrument variables for TB-BMD. LD clumping was conducted with an R square threshold of 0.001. Association data on these instrument variables were extracted from the UK Biobank GWAS summary statistics for circulating SHBG. IVW MR, MR-PRESSO, weighted median MR, MR-Egger regression and MR.RAPS methods using the TwoSampleMR R package were performed as described above.

Multivariable MR was performed to investigate whether BMI or T2DM may partially mediate the causal link between circulating SHBG level and osteoporosis [36]. This method had been described in detail elsewhere [52]. Briefly, this method was based on standard instrumental variable methods which allowed for multiple exposures. It was to estimate the influence of one exposure on outcome conditioning on the other exposures [53].

### Ethics

We conducted this study using published literature and publicly accessible GWAS summary statistics. No primary data collection involving human or animal subjects occurred; therefore, institutional ethical approval was not necessary. All original datasets included in our analysis had obtained their institutional ethics committee approval, with participants providing written informed consent. Our MR analyses were implemented in R (version 3.6.3, R Foundation for Statistical Computing, Vienna, Austria) using the TwoSampleMR package [45]. By default, statistical significance was set at *P* < 0.05 for single test. When multiple testing methods were conducted, significance was determined after Bonferroni correction (*P* < 0.05/N, where N represents the number of tests).

### Data availability

GWAS summary-level data of circulating SHBG level, FN-BMD, LS-BMD, FA-BMD, TB-BMD, BMI and T2DM were publicly available and can be obtained through the links provided in the manuscript. In addition, the datasets used to generate the results in the current study were available in supplementary materials or could receive from the corresponding author on request.

## Results

### Selection of instrumental variables

Detailed information of SNPs (after clumping process) for exposures was listed in Additional File 2. We selected two sets of 11 SNPs and 240 SNPs to serve as instrument variables for circulating SHBG level. In the reverse MR analysis, which was to test whether genetically predicted level of TB-BMD causally influenced circulating SHBG level, we selected 85 SNPs as instrument variables for TB-BMD. Our MR analysis followed the workflow in Fig. 1. F-statistics for all instrument-exposure associations significantly exceeded the threshold of 10, indicating minimal susceptibility of weak instrumental variables bias.

### Two-sample Mendelian randomization estimated the causal link between Circulating SHBG level and BMDs

Tables 1 and 2 present results from the univariate MR analyses of circulating SHBG level and BMDs. In Table 1, IVW, WMM and MR.RAPS using the first set of 11 SNPs suggested a causal effect of increased circulating SHBG level on reduced FA-BMD (IVW estimate: β = −0.39, 95%CI (−0.67, −0.11), *P* = 0.0062; WMM estimate: β =−0.42, 95%CI (−0.74, −0.10), *P* = 0.0096; MR.RAPS estimate: β =−0.38, 95%CI (−0.68, −0.085), *P* = 0.012) and TB-BMD (IVW estimate: β = −0.12, 95%CI (−0.22, −0.022), *P* = 0.016; WMM estimate: β =−0.13, 95%CI (−0.25, −0.0070), *P* = 0.038; MR.RAPS estimate: β =−0.12, 95%CI (−0.22, −0.019), *P* = 0.020). In contrast, univariate MR analyses revealed no causal effect of circulating SHBG level on FN-BMD or LS-BMD. In Table 2, MR analyses using the second set of 240 SNPs suggested a causal effect of increased circulating SHBG level on reduced BMDs of different skeletal sites. Especially, the second MR analyses showed a causal link between increased circulating SHBG level and reduced FN-BMD (MR.RAPS estimate: β = −0.090, 95%CI (−0.14, −0.038), *P* = 0.00067) and LS-BMD (IVW estimate: β = −0.11, 95%CI (−0.16, −0.052), *P* = 1.61E-4; MR.RAPS estimate: β =−0.11, 95%CI (−0.17, −0.057), *P* = 8.64E-5), which was different from the results in Table 1. We considered the difference probably results from the limited number of SNPs in first MR analyses. Potentially owing to the unobvious outlying genetic variants and less precision of MR-Egger regression method, which corrected pleiotropy by allowing the intercept to pass through a value other than zero, we did not find significant causal link between circulating SHBG level and LS-BMD and TB-BMD by this method. Compared with MR-Egger method, WMM MR analysis possessed distinct superiorities, and we found the potential causal link between circulating SHBG level and LS-BMD (β = −0.078, 95%CI (−0.16, 0.0092), *P* = 0.080) and TB-BMD (β = −0.052, 95%CI (−0.11, 0.0069), *P* = 0.083) by using WMM method.

**Table 1:** Mendelian randomization estimates of the association of “circulating SHBG” with “bmds” using the first set of SNPs

		IVW		WMM		MR-Egger						MR.RAPS		MR-PRESSO		F statistic
Disease	SNP	β(95%CI)	*P* value	β(95%CI)	*P* value	Slope(95%CI)	*P* value	Intercept(Se)	*P* value	Cochran Q statistics (df)	*P* value	β(95%CI)	*P* value	β(Se)	*P* value	
FN-BMD	11	−0.024 (−0.19, 0.14)	0.78	−0.045 (−0.20, 0.11)	0.55	−0.065 (−0.41, 0.28)	0.72	0.0020 (0.0077)	0.80	19.67 (9)	0.020	−0.018 (−0.19, 0.16)	0.84	NA	NA	93.3
LS-BMD	10	−0.0040 (−0.23, 0.22)	0.97	0.032 (−0.14, 0.21)	0.72	0.12 (−0.33, 0.58)	0.60	−0.0067 (0.010)	0.53	21.36 (8)	0.0063	−0.0025 (−0.23, 0.23)	0.98	NA	NA	96.5
FA-BMD	11	−0.29 (−0.64, 0.065)	0.11	−0.42 (−0.72, −0.12)	0.0062	−0.61 (−1.30, 0.078)	0.12	0.016 (0.015)	0.31	18.31 (9)	0.032	−0.34 (−0.68, 0.0063)	0.054	−0.39 (0.14)	0.023	93.3
FA-BMD*	10	−0.39 (−0.67, −0.11)	0.0062	−0.42 (−0.74, −0.10)	0.0096	−0.54 (−1.09, 0.072)	0.089	0.0082 (0.013)	0.53	10.32 (8)	0.24	−0.38 (−0.68, −0.085)	0.012	NA	NA	95.4
TB-BMD	8	−0.12 (−0.22, −0.022)	0.016	−0.13 (−0.25, −0.0070)	0.038	−0.13 (−0.32, 0.058)	0.22	0.00047 (0.0043)	0.92	5.49 (6)	0.48	−0.12 (−0.22, −0.019)	0.020	NA	NA	111.0

*IVW* Inverse variance weighting, *WMM* Weighted median, *MR.RAPS* Robust Adjusted Profile Score, *MR-PRESSO* MR-Pleiotropy RESidual Sum and Outlier method, *FN-BMD* Femoral Neck bone mineral density, *LS-BMD* Lumbar Spine bone mineral density, *FA-BMD* Forearm bone mineral density, *TB-BMD* Total body bone mineral density, *β* Beta coefficient, *CI* Confidence interval, *Se* Standard error, *SNP* Single nucleotide polymorphism

*denoted as the MR estimates after removing outliers identified by MR-PRESSO

**Table 2:** Mendelian randomization estimates of the association of “circulating SHBG” with “bmds” using the second set of SNPs

		IVW		WMM		MR-Egger						MR.RAPS		MR-PRESSO		F statistic
Disease	SNP	β(95%CI)	*P* value	β(95%CI)	*P* value	Slope(95%CI)	*P* value	Intercept(Se)	*P* value	Cochran Q statistics (df)	*P* value	β(95%CI)	*P* value	β(Se)	*P* value	
FN-BMD	205	−0.083 (−0.13, −0.034)	0.78	−0.067 (−0.14, 0.0060)	0.072	−0.033 (−0.13, 0.060)	0.49	−0.0017 (0.0014)	0.22	267.06 (203)	0.0017	−0.090 (−0.14, −0.038)	0.00067	NA	NA	124.4
LS-BMD	206	−0.11 (−0.16, −0.047)	0.00044	−0.078 (−0.16, 0.0072)	0.073	0.049 (−0.060, 0.16)	0.38	−0.0054 (0.0016)	0.0013	273.72 (204)	0.00081	−0.11 (−0.17, −0.051)	0.00027	−0.11 (0.029)	0.00021	124.3
LS-BMD*	203	−0.11 (−0.16, −0.052)	1.61E-4	−0.078 (−0.16, 0.0092)	0.080	0.052 (−0.051, 0.16)	0.33	−0.0056 (0.0016)	0.00045	240.16 (201)	0.031	−0.11 (−0.17, −0.057)	8.64E-5	NA	NA	125.4
FA-BMD	209	−0.17 (−0.27, −0.081)	2.35E-4	−0.13 (−0.28, 0.012)	0.072	−0.076 (−0.25, 0.098)	0.39	−0.0034 (0.0026)	0.20	234.19 (207)	0.094	−0.18 (−0.28, −0.091)	9.47E-5	NA	NA	125.0
TB-BMD	228	−0.094 (−0.14, −0.052)	1.55E-5	−0.048 (−0.10, −0.0086)	0.097	−0.030 (−0.11, 0.048)	0.45	−0.0023 (0.0012)	0.055	398.10 (226)	1.24E-11	−0.095 (−0.14, −0.051)	2.17E-5	−0.10 (0.021)	1.41E-6	122.1
TB-BMD*	225	−0.10 (−0.14, −0.062)	7.12E-7	−0.052 (−0.11, 0.0069)	0.083	−0.031 (−0.11, 0.043)	0.41	−0.0025 (0.0011)	0.024	354.05 (223)	5.23E-8	−0.10 (−0.14, −0.059)	2.62E-6	NA	NA	122.8

*IVW* Inverse variance weighting, *WMM* Weighted median, *MR.RAPS* Robust Adjusted Profile Score, *MR-PRESSO* MR-Pleiotropy RESidual Sum and Outlier method, *FN-BMD* Femoral Neck bone mineral density, *LS-BMD* Lumbar Spine bone mineral density, *FA-BMD* Forearm bone mineral density, *TB-BMD* Total body bone mineral density, *β* Beta coefficient, *CI* Confidence interval, *Se* Standard error, *SNP* Single nucleotide polymorphism

*denoted as the MR estimates after removing outliers identified by MR-PRESSO

**Fig. 2:**
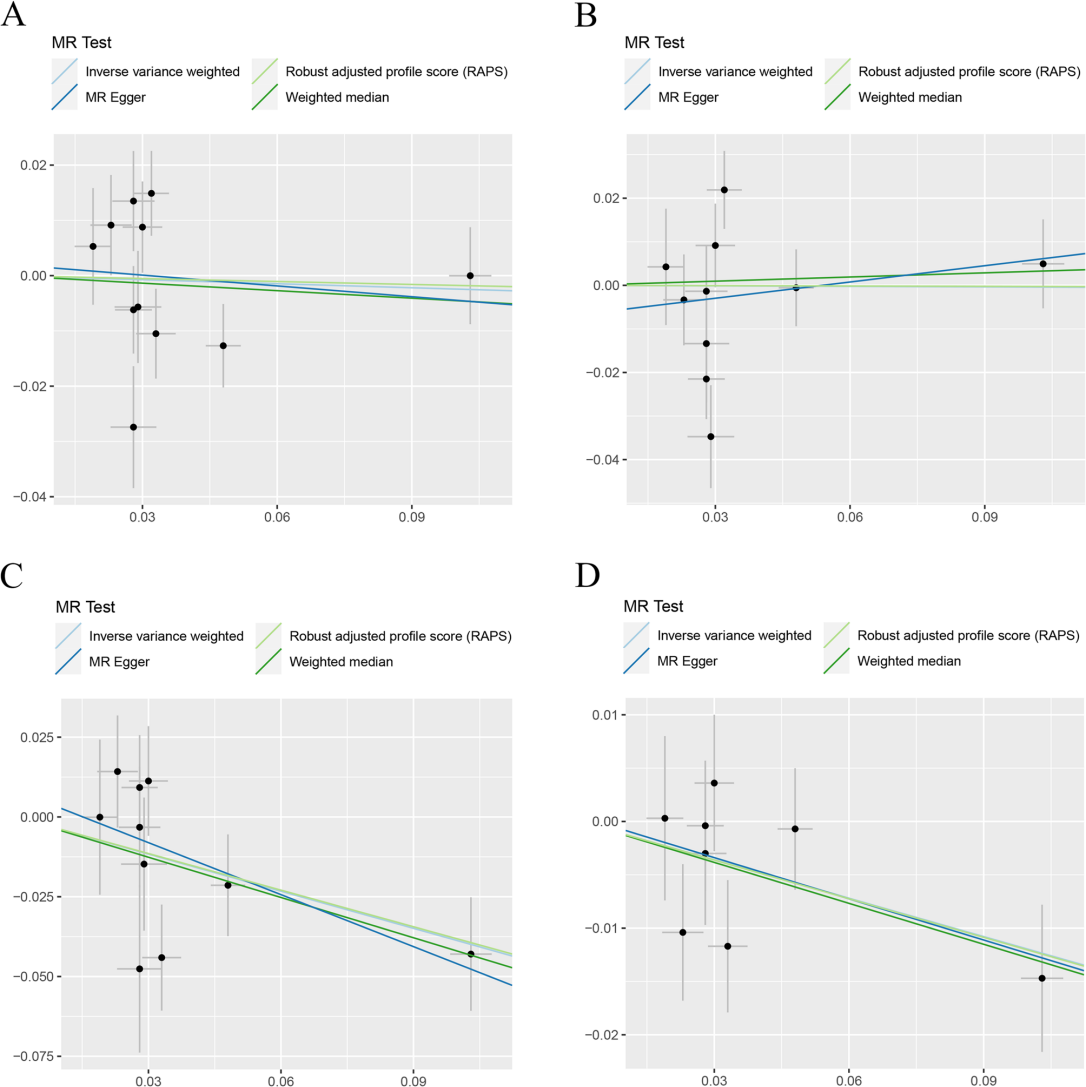
Scatter plots for MR analyses of the causal link between circulating SHBG level and **A** FN-BMD **B** LS-BMD **C** FA-BMD **D** TB-BMD using first set of SNPs. Analyses were conducted using the conventional IVW, MR.RAPS, MR-Egger and WMM methods. The slope of each line corresponding to the estimated MR effect per method

**Fig. 3:**
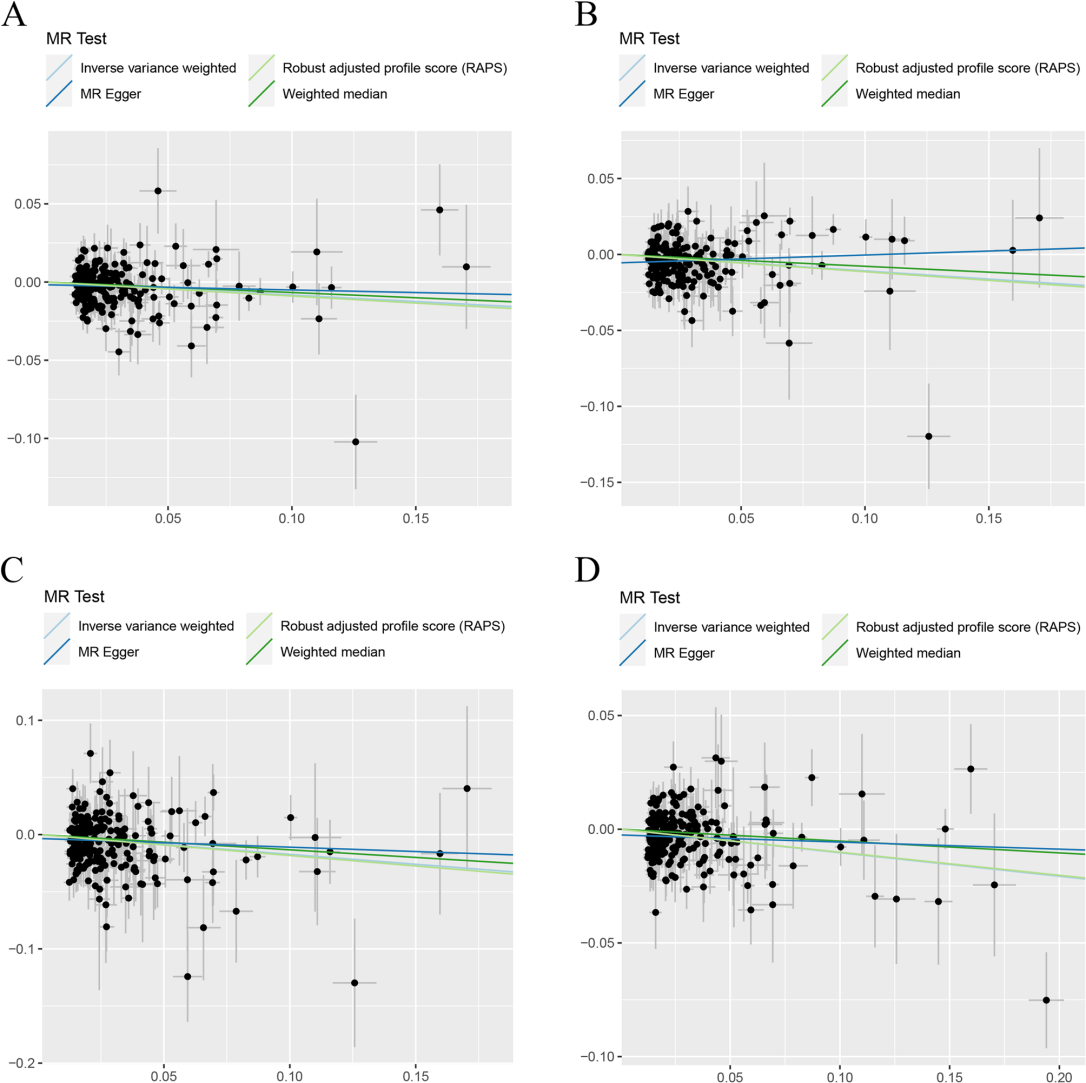
Scatter plots for MR analyses of the causal link between circulating SHBG level and **A** FN-BMD **B** LS-BMD **C** FA-BMD **D** TB-BMD using second set of SNPs. Analyses were conducted using the conventional IVW, MR.RAPS, MR-Egger and WMM methods. The slope of each line corresponding to the estimated MR effect per method

Heterogeneity tests highlighted the existence of significant heterogeneity in most groups, even though we removed the outliers identified by MR-PRESSO. In Tables 1 and 2, our analyses suggested no significant evidence of horizontal pleiotropy (as indicated by MR-Egger regression intercept close to zero, with a *P* value larger than 0.05) in some heterogeneous groups. This suggested potential cancellation of pleiotropic effects when the estimates were combined together in meta-analysis/Egger regression. In Table 2, the genetically instrumented circulating SHBG level was causally associated with LS-BMD and TB-BMD using IVM method and MR.RAPS method. Although the two groups exhibited significant heterogeneity and horizontal pleiotropy (P for MR-Egger intercept < 0.05), MR-RAPS estimates, robust to systematic and idiosyncratic pleiotropy, indicated convincible and reliable causal link. Scatter plots (Figs. 2 and 3) displayed the estimated effect sizes of SNPs on exposure (SHBG) and BMDs outcomes. The funnel plots (Additional File 3: Figure S1 and Additional File 3: Figure S2) provided indication of whether there existed directional horizontal pleiotropy for each outcome. Leave-one-out analysis (Additional File 3: Figure S3) identified potentially influential SNPs (among the first set of 11 SNPs) driving the causal link between circulating SHBG level and FA-BMD and TB-BMD. Additional File 3: Figure S4 demonstrated that no potentially influential SNPs (among the second set of 240 SNPs) driving the causal link between circulating SHBG level and BMDs was detected.

### Causal link between Circulating SHBG level and BMDs were not mediated through BMI or T2DM

Searching through phenoscanner (http://www.phenoscanner.medschl.cam.ac.uk/) [54], we found that some of the selected genetic variants for circulating SHBG level were significantly associated with BMI and T2DM. Thus, we took steps to examine the possibility that BMI and T2DM mediate the causal effect of circulating SHBG level on BMDs. In this part, our MR analyses also proceeded as Fig. 1. Tables 3 and 4 presented results from the univariate MR analyses of circulating SHBG level with BMI and T2DM. In Table 3, univariate MR analyses revealed no evidence for a causal effect of circulating SHBG level on BMI or T2DM using the limited number of SNPs (11 SNPs). However, in Table 4, MR analyses with the second set of 240 SNPs demonstrated a causal link between increased circulating SHBG level and decreased BMI and decreased risk of T2DM using IVW, WMM and MR.RAPS methods. We did not find significant causal link between circulating SHBG level and BMI or T2DM using MR-Egger regression method, potentially owing to the existence of unobvious outlying genetic variants and lack precision of this method.

**Table 3:** Mendelian randomization estimates of the association of “circulating SHBG” with “BMI” and “T2DM” using the first set of SNPs

		IVW		WMM		MR-Egger						MR.RAPS		MR-PRESSO		F statistics
Outcome	SNP	β(95%CI)	*P* value	β(95%CI)	*P* value	Slope(95%CI)	*P* value	Intercept(Se)	*P* value	Cochran Q statistics (df)	*P* value	β(95%CI)	*P* value	β(Se)	*P* value	
BMI	8	0.045 (−0.024, 0.11)	0.20	0.053 (−0.022, 0.13)	0.17	0.13 (0.010, 0.25)	0.078	−0.0047 (0.0027)	0.14	1.78 (6)	0.94	0.047 (−0.024, 0.12)	0.19	NA	NA	93.15
T2DM	10	−0.17 (−0.50, 0.17)	0.33	−0.21 (−0.54, −0.11)	0.20	−0.43 (−1.07, 0.22)	0.23	0.013 (0.014)	0.38	13.32 (8)	0.10	−0.20 (−0.50, 0.097)	0.18	NA	NA	95.4

*IVW* Inverse variance weighting, *WMM* Weighted median, *MR.RAPS* Robust Adjusted Profile Score, *MR-PRESSO* MR-Pleiotropy RESidual Sum and Outlier method, *BMI* Body mass index, *T2DM* Type 2 diabetes mellitus, *β* Beta coefficient, *CI* Confidence interval, *Se* Standard error, *SNP *Single nucleotide polymorphism

**Table 4:** Mendelian randomization estimates of the association of “circulating SHBG” with “BMI” and “T2DM” using the second set of SNPs

		IVW		WMM		MR-Egger						MR.RAPS		MR-PRESSO		F statistics
Outcome	SNP	β(95%CI)	*P* value	β(95%CI)	*P* value	Slope(95%CI)	*P* value	Intercept(Se)	*P* value	Cochran Q statistics (df)	*P* value	β(95%CI)	*P* value	β(Se)	*P* value	
BMI	154	−0.022 (−0.056, 0.012)	0.20	−0.062 (−0.11, −0.017)	0.0076	−0.039 (−0.10, 0.026)	0.24	0.00056 (0.00095)	0.56	308.60 (153)	1.01E-12	−0.027 (−0.063, 0.0080)	0.13	−0.027 (0.017)	0.12	117.0
BMI*	151	−0.035 (−0.068, −0.0023)	0.036	−0.062 (−0.11, −0.018)	0.0063	−0.055 (−0.12, 0.0070)	0.084	0.00068 (0.00091)	0.46	272.86 (149)	2.57E-9	−0.038 (−0.072, −0.0041)	0.028	NA	NA	115.7
T2DM	222	−0.26 (−0.39, −0.14)	0.000034	−0.20 (−0.36, −0.038)	0.015	−0.062 (−0.29, 0.16)	0.59	−0.0073 (0.0035)	0.038	431.83 (220)	6.43E-16	−0.25 (−0.37, −0.12)	0.00012	−0.24 (0.059)	9.98E-5	126.5
T2DM*	216	−0.26 (−0.37, −0.14)	1.48E-5	−0.21 (−0.38, −0.036)	0.018	−0.059 (−0.27, 0.15)	0.58	−0.0070 (0.0032)	0.030	341.32 (214)	6.94E-8	−0.24 (−0.36, −0.12)	6.24E-5	NA	NA	123.9

*IVW* Inverse variance weighting, *WMM* Weighted median, *MR.RAPS* Robust Adjusted Profile Score, *MR-PRESSO* MR-Pleiotropy RESidual Sum and Outlier method, *BMI* Bdy mass index, *T2DM* Type 2 diabetes mellitus, *β* Beta coefficient, *CI* Confidence interval, *Se* Standard error, *SNP* Single nucleotide polymorphism

*denoted as the MR estimates after removing outliers identified by MR-PRESSO

**Fig. 4:**
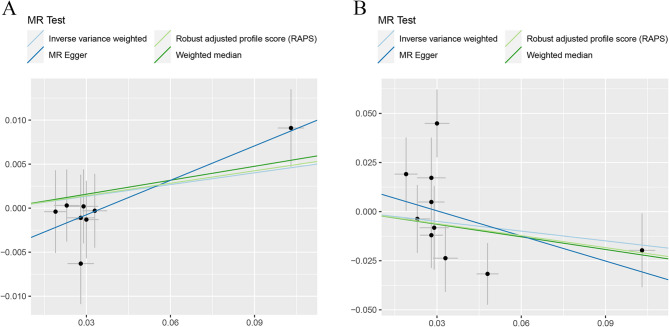
Scatter plots for MR analyses of the causal link between circulating SHBG level and **A** BMI and **B** risk of T2DM using first set of SNPs. Analyses were conducted using the conventional IVW, MR.RAPS, MR-Egger and WMM methods. The slope of each line corresponding to the estimated MR effect per method

**Fig. 5:**
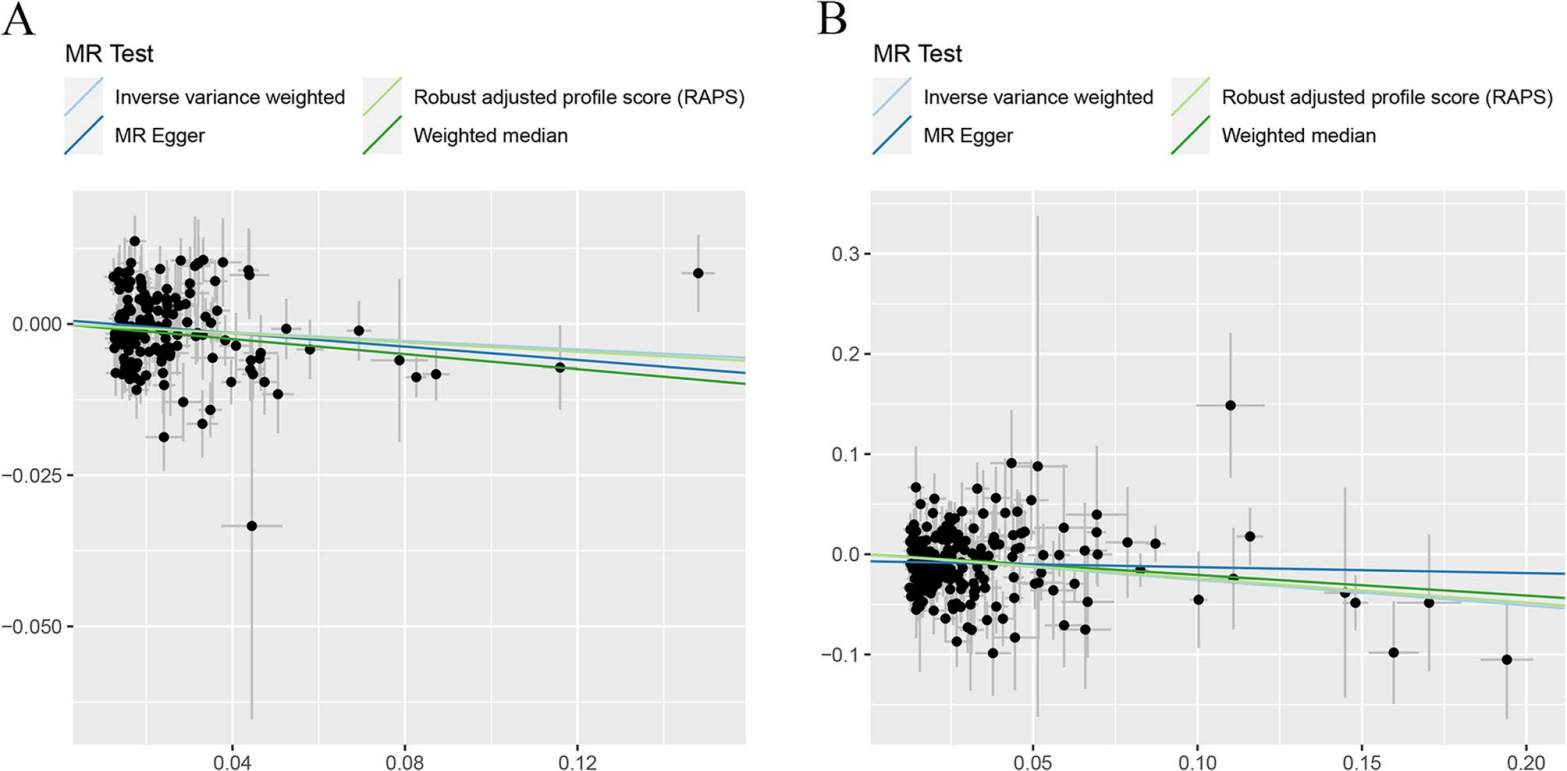
Scatter plots for MR analyses of the causal link between circulating SHBG level and **A** BMI and **B** risk of T2DM using second set of SNPs. Analyses were conducted using the conventional IVW, MR.RAPS, MR-Egger and WMM methods. The slope of each line corresponding to the estimated MR effect per method

In Table 4, both groups of BMI and T2DM presented significant heterogeneity and groups of T2DM accompanied with significant horizontal pleiotropy (P for MR-Egger intercept < 0.05). Despite this, MR-RAPS estimates (MR.RAPS estimate: β = −0.24, 95%CI ((−0.36, −0.12), *P* = 6.24E-5) demonstrated a reliable causal relationship between elevated circulating SHBG level and reduced T2DM risk. The scatter plots (Fig. 4 and Fig. 5) displayed the estimated effect sizes of the SNPs on both the exposure (SHBG) and outcomes (BMI and T2DM). The funnel plots (Additional File 3: Figure S5 and Additional File 3: Figure S6) provided indication of whether there existed directional horizontal pleiotropy for each outcome. Leave-one-out analysis (Additional File 3: Figure S7) demonstrated that there was no potentially influential SNPs (among the set of 11 SNPs) driving the causal link between circulating SHBG level and BMI or T2DM. However, Additional File 3: Figure S8 identified potentially influential SNPs (among the set of 240 SNPs) driving the causal link between circulating SHBG level and BMI but not T2DM, necessitating cautious interpretation of this specific causal link.

We performed univariate MR analyses and found the potential causal effect of circulating SHBG level on BMI and risk of T2DM in Table 4, which was consistent with published articles [33, 55]. Previous publications had shown BMI and T2DM had positively causal link with BMD [56, 57]. Thus, we inferred BMI and T2DM had the probability to work as mediators between circulating SHBG level and BMDs. We performed multivariable MR analyses for each pair of (BMI and SHBG) and (T2DM and SHBG) to investigate whether either of these two traits (BMI and T2DM) might mediate the causal effect of circulating SHBG level on BMDs. The multivariable MR analysis allowed us to estimate and test the direct effect of circulating SHBG level on BMDs independent of the complex traits. In Table 5, results of multivariable MR analysis demonstrated the causal effect of circulating SHBG on BMDs was independent of BMI and T2DM. In other words, the causal effects of circulating SHBG level on the BMDs were not obviously mediated through BMI or T2DM.

**Table 5:** Multivariable Mendelian randomization estimates of the causal association of “circulating SHBG” on “BMD” independent of BMI and T2DM

Exposure	FN-BMD		LS-BMD		FA-BMD		TB-BMD	
	β(Se)	*P* value	β(Se)	*P* value	β(Se)	*P* value	β(Se)	*P* value
SHBG and BMI	Snp = 147 −0.099 (0.029)	0.00062	Snp = 147 −0.10 (0.034)	0.0019	Snp = 149 −0.16 (0.057)	0.0057	Snp = 159 −0.098 (0.031)	0.0014
SHBG and T2DM	Snp = 201 −0.070 (0.026)	0.0066	Snp = 201 −0.091 (0.032)	0.0041	Snp = 204 −0.16 (0.048)	0.0010	Snp = 227 −0.082 (0.026)	0.0014

*FN-BMD* Femoral Neck bone mineral density, *LS-BMD* Lumbar Spine bone mineral density, *FA-BMD* Forearm bone mineral density, *TB-BMD* Total body bone mineral density, *β* Beta coefficient, *Se* Standard error, *SNP* Single nucleotide polymorphism, *BMI* Body mass index, *T2DM* Type 2 diabetes mellitus

### Bidirectional MR demonstrated no reverse causal effect of TB-BMD on Circulating SHBG level

**Fig. 6:**
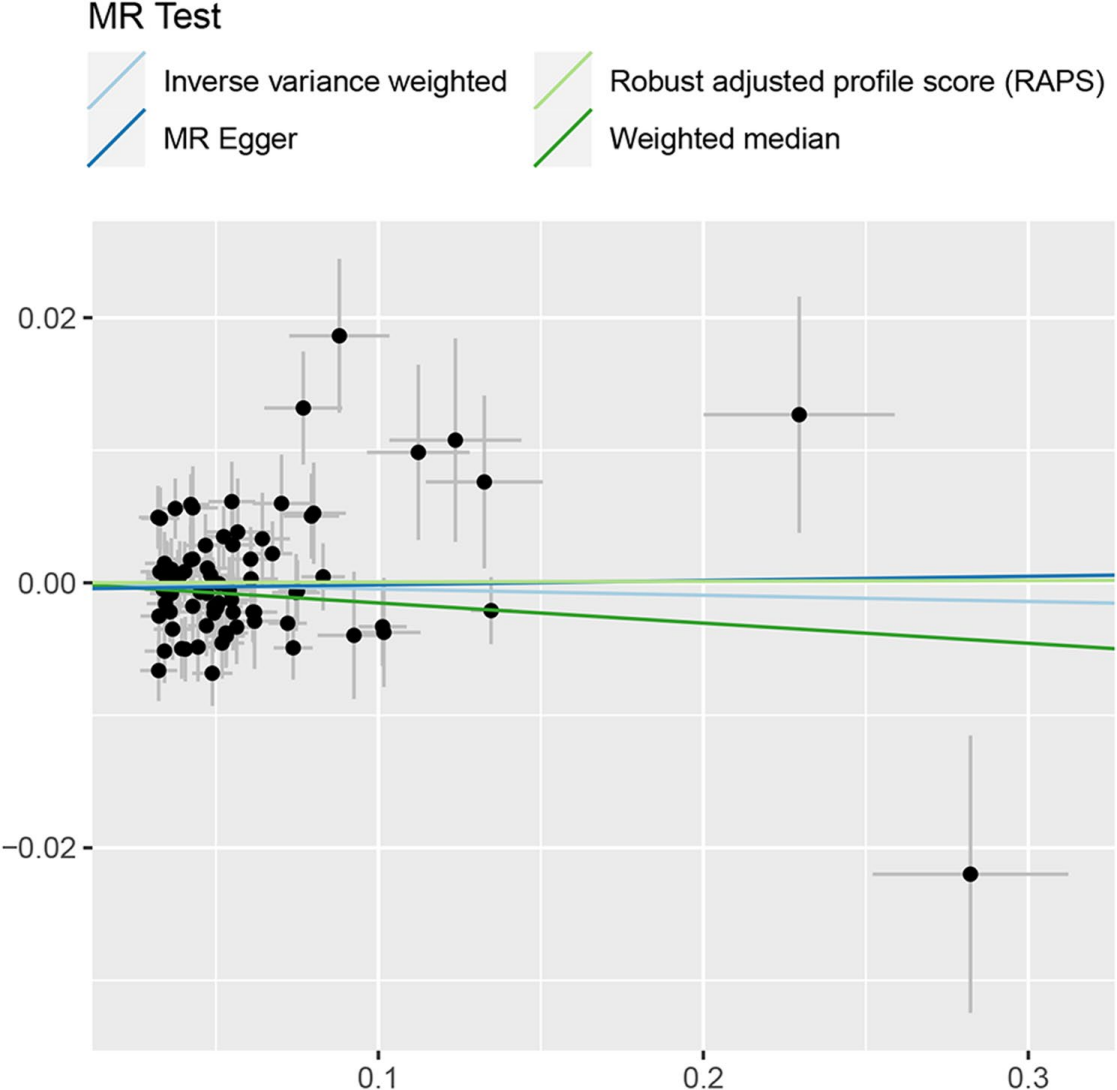
Scatter plots for bidirectional MR analysis of the causal link between TB-BMD level and circulating SHBG level. Analyses were conducted using the conventional IVW, MR.RAPS, MR-Egger and WMM methods. The slope of each line corresponding to the estimated MR effect per method

To guard against the possibility that our results were driven by reverse causality, we performed reverse MR analysis testing whether TB-BMD causally influences circulating SHBG level. After LD clumping, 85 SNPs showed robustly association with TB-BMD. As presented in Table 6, IVW, WMM, MR-Egger regression and MR.RAPS analyses consistently indicated no significant reverse causal associations between TB-BMD and circulating SHBG level. Cochrane Q statistics suggested substantial heterogeneity in the analysis, though horizontal pleiotropy was insignificant (P for MR-Egger intercept > 0.05). This pattern suggests potential cancellation of horizontal pleiotropy when the estimates were combined together in meta-analysis/Egger regression. The scatter plot, funnel plot and Plot of leave-one-out analysis for the bidirectional MR were presented in Fig. 6, Additional File 3: Figure S9 and Additional File 3: Figure S10 respectively.

**Table 6:** Bidirectional MR estimating the reverse causal effect of “TB-BMD” on Circulating SHBG level

		IVW		WMM		MR-Egger						MR.RAPS		MR-PRESSO		F statistic
Exposure	SNP	β(95%CI)	P value	β(95%CI)	P value	Slope(95%CI)	P value	Intercept(Se)	P value	Cochran Q statistics (df)	P value	β(95%CI)	P value	β(Se)	P value	
TB-BMD	78	−0.0047 (−0.019, 0.010)	0.53	−0.015 (−0.034, 0.0035)	0.11	0.0032 (−0.035, 0.042)	0.87	−0.00047 (0.0011)	0.66	141.53 (76)	7.59E-6	0.00055 (−0.018, 0.019)	0.95	NA	NA	76.0

*IVW* Inverse variance weighting, *WMM* Weighted median, *MR.RAPS* Robust Adjusted Profile Score, *MR-PRESSO* MR-Pleiotropy RESidual Sum and Outlier method, *TB-BMD* Total body bone mineral density, *β* Beta coefficient, *CI* Confidence interval, *Se* Standard error, *SNP* Single nucleotide polymorphism

## Discussion

We conducted a large MR study using the publicly available summary statistics of GWAS data for circulating SHBG level and BMDs. In this two-sample MR analysis, we determined a causal effect of genetically predicted high circulating SHBG level on decreased FA-BMD and TB-BMD but not on FN-BMD or LS-BMD, when using the first set of 11 SNPs to instrument circulating SHBG level. When using the second set of 240 SNPs to instrument circulating SHBG level, we got the causal effect of increased circulating SHBG level on decreased BMDs of different skeletal sites (including FN-BMD, LS-BMD, FA-BMD and TB-BMD). We considered the difference probably resulted from the limited number of SNPs in first MR analysis, causing low precision. In second MR analysis, we utilized hundreds of stringently selected weak SNPs as the instrument variables, it substantially decreased the variance of the estimator. We thought the results of second MR analysis were more robust and reliable than the first one. We performed univariate and multivariable MR analyses to determine whether BMI and T2DM had the probability to work as mediators or confounders in the causal effect of circulating SHBG level on BMDs. The results demonstrated the causal link between increased circulating SHBG level and decreased BMDs was independent of the potential horizontal pleiotropy acting via BMI or T2DM. We also performed reverse MR analysis to guard against the possibility that our results were driven by the potential causal effects of decreased BMD on increased circulating SHBG level. We comprehensively summarized all the MR results and concluded that there was a causal link between increased circulating SHBG level and decreased BMD of different skeletal sites. In future researches, the role of circulating SHBG in osteoporosis could be prioritized aiming to investigate its biological effects on bone health and underlying mechanism, finally to develop targets for prevention and therapeutic intervention.

Our results were somewhat consistent with the Mendelian randomization phenome-wide association study (MR-pheWAS), which used data from a large-scale cohort study (UK Biobank) and indicated the associations of increased natural log SHBG with heel bone ultrasound attenuation (in the right foot) [58]. However, the MR-pheWAS didn’t assess causal effect of circulating SHBG level on FN-BMD, LS-BMD, FA-BMD and TB-BMD, these skeletal sites hold distinct clinical relevance. Femoral neck, lumbar spine and forearm are standard DXA measurement skeletal sites for osteoporosis assessment in postmenopausal women and men ≥ 50 years. TB-BMD measurement provides unbiased longitudinal assessment of BMD variation in the same skeletal site across the lifespan [35]. Therefore, our MR analysis specifically targeted these anatomically distinct BMD measures (including FN-BMD, LS-BMD, FA-BMD and TB-BMD).

The relationships between circulating SHBG level and risk of osteoporosis weren’t consistent in published studies. Multiple observational studies reported significant associations between circulating SHBG levels and osteoporosis or fracture risk [10, 19, 59], aligning with our MR study findings. However, Eriksson et al. presented contradictory evidence, human genetic variants associated with elevated SHBG correlated with higher BMD, and male murine models overexpressing SHBG showed increased femoral cortical bone mineral content. This suggests SHBG may exert bone-protective effects in specific biological contexts [25]. A few observational publications demonstrated no significant effect of SHBG concentration on BMD after multivariate analysis [20–24]. Discrepancies between our MR results and certain observational studies might be explained as follows: firstly, epidemiological findings are frequently constrained by limited sample sizes. MR analysis enabled the use of very large publicly available GWAS data for both risk factor “exposures” and disease “outcomes”. Secondly, even though the epidemiological observational studies showed no significant association between circulating SHBG level and BMD under the adjustment for many main components, they might be affected by the other related factors. Thirdly, the results of our MR analysis might be biased by horizontal pleiotropy [60]. It was impossible to completely fulfill the three specific assumptions of MR analysis. However, we deemed the possibility that pleiotropy significantly biased the results was tiny in this analysis. Because we implemented multiple robustness measures (e.g., MR-PRESSO outlier test, MR.RAPS method, multivariable MR analysis and sensitivity analyses) to mitigate the influence of horizontal pleiotropy and ensure robust and reliable causal inference.

Our research determined the causal link between circulating SHBG, which could either work as sex hormone transporter or function beyond the role of sex hormone, and risk of osteoporosis under the two-sample MR analysis framework. It overcame many of the typical pitfalls present in observational studies and RCT. The F statistics substantially exceeded 10, hinting the small possibility of weak instrumental variables bias [43]. Power estimates (calculated via mRnd: https://shiny.cnsgenomics.com/mRnd/) approached 1.0 for all analyses, indicating minimal probability of making a Type II error. We performed MR-PRESSO outlier test to identify and remove outlier variants. Multivariable MR analysis was performed to control for potential horizontal pleiotropy acting via BMI or T2DM. Bidirectional MR analysis was performed to exclude the reverse causality. Several robust methods for MR analysis were adopted in our manuscript, which could provide reliable inferences when some genetic variants violated the instrument variables assumptions. Especially, MR.RAPS which was robust to both systematic and idiosyncratic pleiotropy was implemented to obtain a robust inference for our MR analysis with many weak IVs. Besides, we utilized four groups of BMD summary GWAS data (FN-BMD, LS-BMD, FA-BMD, and TB-BMD) and two groups of exposure summary GWAS data within our two-sample MR framework. Lastly, all GWAS summary statistics we downloaded for circulating SHBG and BMDs were derived exclusively from European-ancestry populations and adjusted for principal components, enhancing internal validity through population homogeneity. All these above were the highlights of our research, serving to reduce potential bias and enhance the credibility of our results. Nevertheless, several limitations warrant consideration in our analysis. First, two-sample MR analysis ideally requires non-overlapping samples between exposure and outcome studies. We were unable to estimate the degree of potential sample overlap in the study. However, bias from such overlap could be mitigated by our use of strong instruments (e.g. F statistic much greater than 10) [61]. Second, the summary GWAS data derived exclusively from individuals of European descent limits the generalizability of our findings. Therefore, caution is advised when applying our conclusions to racially and ethnically diverse populations. Third, despite steps taken to minimize its influence, we cannot exclude the possibility that horizontal pleiotropy affected our results. Fourth, the use of multiple MR methods, each with its own inherent strengths and limitations based on different assumptions, increases the potential for inconsistent or contradictory results, potentially obscuring the conclusions. Fifth, significant heterogeneity persisted in some analyses even after removing outliers identified by MR-PRESSO. Sixth, except for MR-PRESSO outlier test and multivariable MR analysis, we did not take the other measures (e.g., searching through Ensembl project or Phenoscanner database) to identify and exclude genetic variants associated with potential confounding factors. Seventh, MR study performed by other groups pointed out that SHBG was closely linked to osteoporosis risk specifically in female population but not in males [62]. An inverse association with total-body BMD was observed in individuals over 45 years in age-stratified MR analyses [63]. We did not repeat sex-stratified or age-group stratified MR analyses to verify the results in our manuscript.

## Conclusion

In this study, we determined a causal effect of genetically predicted high circulating SHBG level on decreased BMDs of different skeletal sites. Updated MR analysis incorporating populations from different races may be warranted to confirm our findings in the future. Further exploration of the potential mediator between circulating SHBG concentration and osteoporosis also may be meaningful. Foremost, our result can be helpful for researchers to develop new treatment target for osteoporosis, or reminding clinicians of taking measures and concerted efforts to prevent or intervene with bone loss when patients are diagnosed as high circulating SHBG level.

## Supplementary Information

Additional file 1. Characteristics of the selected 16 SNPs for circulating SHBG concentration.

Additional file 2. Detailed information of LD-independent SNPs (after clumping process) for exposures.

Additional file 3. Figure S1. Funnel plots for MR analyses assessing the causal effect of circulating SHBG on BMDs using the first set of SNPs (A) FN-BMD (B) LS-BMD (C) FA-BMD (D) TB-BMD. Figure S2. Funnel plots for MR analyses assessing the causal effect of circulating SHBG on BMDs using the second set of SNPs (A) FN-BMD (B) LS-BMD (C) FA-BMD (D) TB-BMD. Figure S3. Plots of “leave-one-out” analyses for MR analyses assessing the causal effect of circulating SHBG on BMDs using the first set of SNPs (A) FN-BMD (B) LS-BMD (C) FA-BMD (D) TB-BMD. Figure S4. Plots of “leave-one-out” analyses for MR analyses assessing the causal effect of circulating SHBG on BMDs using the second set of SNPs (A) FN-BMD (B) LS-BMD (C) FA-BMD (D) TB-BMD. Figure S5. Funnel plots for MR analyses assessing the causal effect of circulating SHBG on BMI and T2DM using the first set of SNPs (A) BMI (B) T2DM. Figure S6. Funnel plots for MR analyses assessing the causal effect of circulating SHBG on BMI and T2DM using the second set of SNPs (A) BMI (B) T2DM. Figure S7. Plots of “leave-one-out” analyses for MR analyses assessing the causal effect of circulating SHBG on BMI and T2DM using the first set of SNPs (A) BMI (B) T2DM. Figure S8. Plots of “leave-one-out” analyses for MR analyses assessing the causal effect of circulating SHBG on BMI and T2DM using the second set of SNPs (A) BMI (B) T2DM. Figure S9. Funnel plots for the reverse MR analyses assessing the causal effect of TB-BMD on circulating SHBG level. Figure S10. Plots of “leave-one-out” analyses for the reverse MR analyses assessing the causal effect of TB-BMD on circulating SHBG level.

## Data Availability

Data is provided within the manuscript or supplementary information files; https://gwas.mrcieu.ac.uk/, http://www.gefos.org/, http://www.type2diabetesgenetics.org/, http://cg.bsc.es/70kfort2d/.

## Abbreviations

BMD: Bone mineral density
DXA: Dual-energy X-ray absorptiometry
BMI: Body mass index
T: Testosterone
E2: Estradiol
SHBG: Sex hormone-binding globulin
RCT: Randomized controlled trial
MR: Mendelian randomization
GWAS: Genome-wide association study
T2DM: Type 2 diabetes
SNP: Single-nucleotide polymorphism
IV: Instrument variable
GEFOS: Genetic factors for osteoporosis consortium
FN-BMD: Femoral neck bone mineral density
LS-BMD: Lumbar spine bone mineral density
FA-BMD: Forearm bone mineral density
TB-BMD: Total body-bone mineral density
LD: Linkage disequilibrium
IVW: Inverse variance weighting
MR-PRESSO: MR-pleiotropy residual sum and Outlier method
MBE: Mode based estimate method
WMM: Weighted median method
MR.RAPS: Robust adjusted profile score
InSIDE: Instrument strength independent of direct effect
MR-pheWAS: Mendelian randomization phenome-wide association study
