# Supplementary figures and images for: Mendelian randomization study reveals the causal effect of sex hormone binding globulin on osteoporosis
Source: BMC Musculoskelet Disord. 2025 Jul 26;26:713. doi: 10.1186/s12891-025-08956-7 (PMC12297757; doi:10.1186/s12891-025-08956-7)

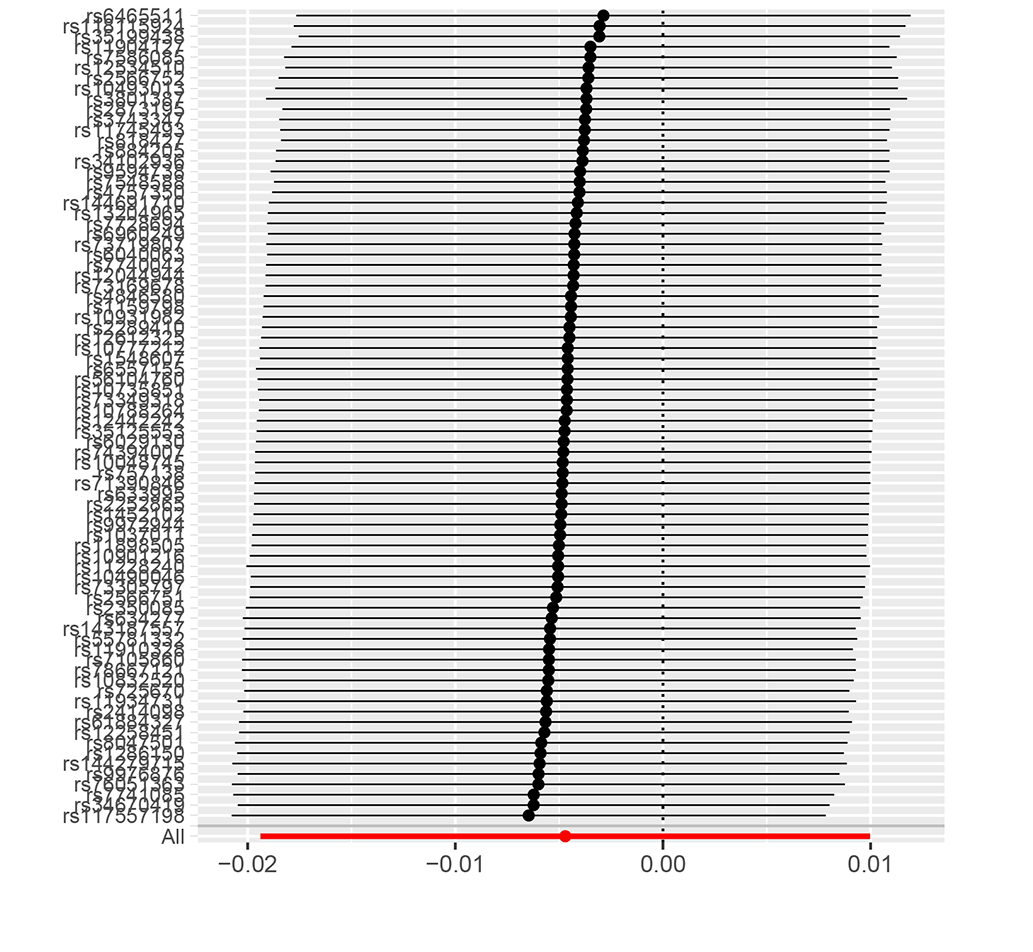

Supplement: Supplementary file 3 — Additional file 3. Figure S1. Funnel plots for MR analyses assessing the causal effect of circulating SHBG on BMDs using the first set of SNPs (A) FN-BMD (B) LS-BMD (C) FA-BMD (D) TB-BMD. Figure S2. Funnel plots for MR analyses assessing the causal effect of circulating SHBG on BMDs using the second set of SNPs (A) FN-BMD (B) LS-BMD (C) FA-BMD (D) TB-BMD. Figure S3. Plots of “leave-one-out” analyses for MR analyses assessing the causal effect of circulating SHBG on BMDs using the first set of SNPs (A) FN-BMD (B) LS-BMD (C) FA-BMD (D) TB-BMD. Figure S4. Plots of “leave-one-out” analyses for MR analyses assessing the causal effect of circulating SHBG on BMDs using the second set of SNPs (A) FN-BMD (B) LS-BMD (C) FA-BMD (D) TB-BMD. Figure S5. Funnel plots for MR analyses assessing the causal effect of circulating SHBG on BMI and T2DM using the first set of SNPs (A) BMI (B) T2DM. Figure S6. Funnel plots for MR analyses assessing the causal effect of circulating SHBG on BMI and T2DM using the second set of SNPs (A) BMI (B) T2DM. Figure S7. Plots of “leave-one-out” analyses for MR analyses assessing the causal effect of circulating SHBG on BMI and T2DM using the first set of SNPs (A) BMI (B) T2DM. Figure S8. Plots of “leave-one-out” analyses for MR analyses assessing the causal effect of circulating SHBG on BMI and T2DM using the second set of SNPs (A) BMI (B) T2DM. Figure S9. Funnel plots for the reverse MR analyses assessing the causal effect of TB-BMD on circulating SHBG level. Figure S10. Plots of “leave-one-out” analyses for the reverse MR analyses assessing the causal effect of TB-BMD on circulating SHBG level. [file 12891_2025_8956_MOESM3_ESM.zip › Additional File 3 Figure S10.tif]

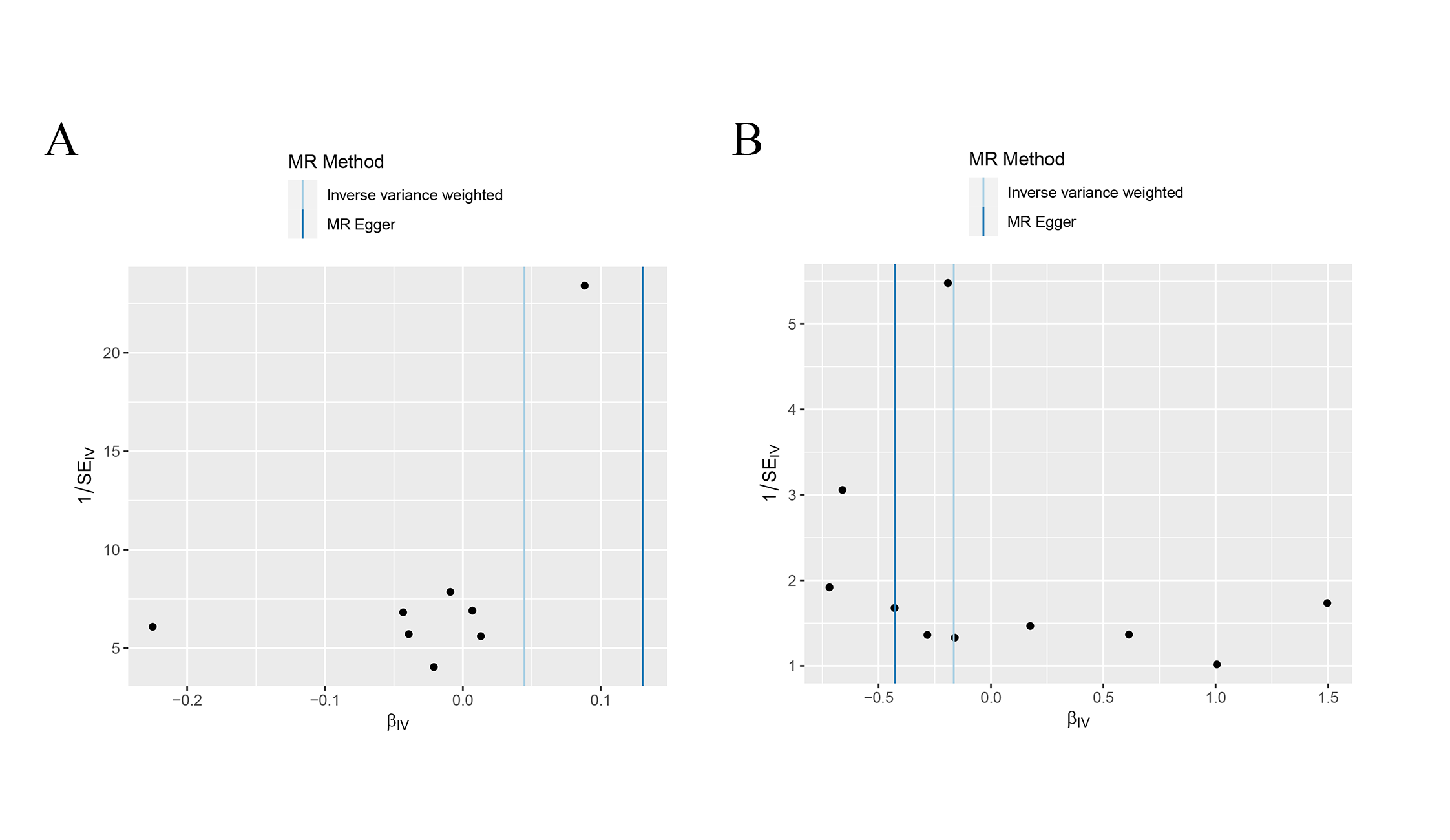

Supplement: Supplementary file 3 — Additional file 3. Figure S1. Funnel plots for MR analyses assessing the causal effect of circulating SHBG on BMDs using the first set of SNPs (A) FN-BMD (B) LS-BMD (C) FA-BMD (D) TB-BMD. Figure S2. Funnel plots for MR analyses assessing the causal effect of circulating SHBG on BMDs using the second set of SNPs (A) FN-BMD (B) LS-BMD (C) FA-BMD (D) TB-BMD. Figure S3. Plots of “leave-one-out” analyses for MR analyses assessing the causal effect of circulating SHBG on BMDs using the first set of SNPs (A) FN-BMD (B) LS-BMD (C) FA-BMD (D) TB-BMD. Figure S4. Plots of “leave-one-out” analyses for MR analyses assessing the causal effect of circulating SHBG on BMDs using the second set of SNPs (A) FN-BMD (B) LS-BMD (C) FA-BMD (D) TB-BMD. Figure S5. Funnel plots for MR analyses assessing the causal effect of circulating SHBG on BMI and T2DM using the first set of SNPs (A) BMI (B) T2DM. Figure S6. Funnel plots for MR analyses assessing the causal effect of circulating SHBG on BMI and T2DM using the second set of SNPs (A) BMI (B) T2DM. Figure S7. Plots of “leave-one-out” analyses for MR analyses assessing the causal effect of circulating SHBG on BMI and T2DM using the first set of SNPs (A) BMI (B) T2DM. Figure S8. Plots of “leave-one-out” analyses for MR analyses assessing the causal effect of circulating SHBG on BMI and T2DM using the second set of SNPs (A) BMI (B) T2DM. Figure S9. Funnel plots for the reverse MR analyses assessing the causal effect of TB-BMD on circulating SHBG level. Figure S10. Plots of “leave-one-out” analyses for the reverse MR analyses assessing the causal effect of TB-BMD on circulating SHBG level. [file 12891_2025_8956_MOESM3_ESM.zip › Additional File 3 Figure S5.tif]

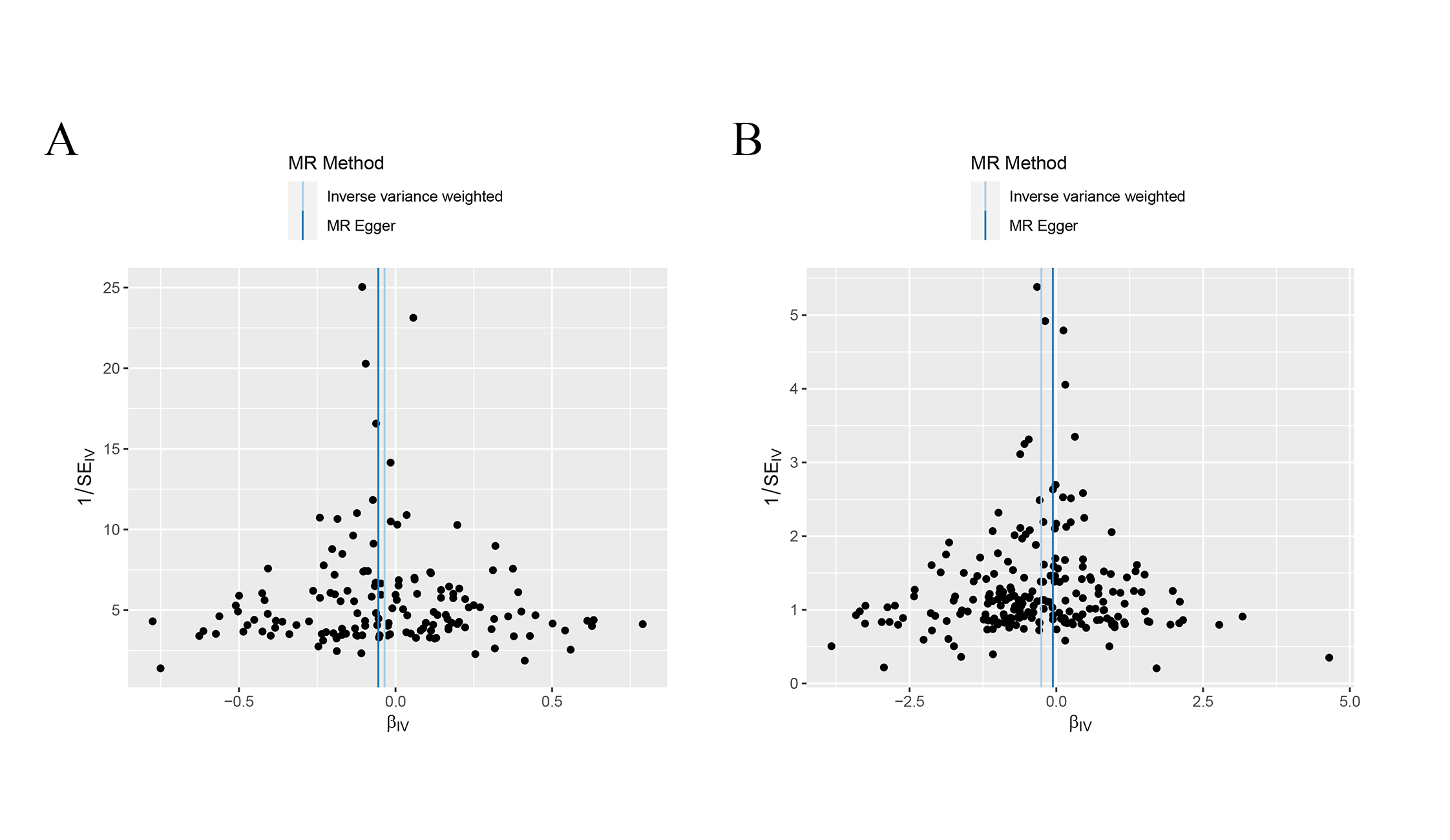

Supplement: Supplementary file 3 — Additional file 3. Figure S1. Funnel plots for MR analyses assessing the causal effect of circulating SHBG on BMDs using the first set of SNPs (A) FN-BMD (B) LS-BMD (C) FA-BMD (D) TB-BMD. Figure S2. Funnel plots for MR analyses assessing the causal effect of circulating SHBG on BMDs using the second set of SNPs (A) FN-BMD (B) LS-BMD (C) FA-BMD (D) TB-BMD. Figure S3. Plots of “leave-one-out” analyses for MR analyses assessing the causal effect of circulating SHBG on BMDs using the first set of SNPs (A) FN-BMD (B) LS-BMD (C) FA-BMD (D) TB-BMD. Figure S4. Plots of “leave-one-out” analyses for MR analyses assessing the causal effect of circulating SHBG on BMDs using the second set of SNPs (A) FN-BMD (B) LS-BMD (C) FA-BMD (D) TB-BMD. Figure S5. Funnel plots for MR analyses assessing the causal effect of circulating SHBG on BMI and T2DM using the first set of SNPs (A) BMI (B) T2DM. Figure S6. Funnel plots for MR analyses assessing the causal effect of circulating SHBG on BMI and T2DM using the second set of SNPs (A) BMI (B) T2DM. Figure S7. Plots of “leave-one-out” analyses for MR analyses assessing the causal effect of circulating SHBG on BMI and T2DM using the first set of SNPs (A) BMI (B) T2DM. Figure S8. Plots of “leave-one-out” analyses for MR analyses assessing the causal effect of circulating SHBG on BMI and T2DM using the second set of SNPs (A) BMI (B) T2DM. Figure S9. Funnel plots for the reverse MR analyses assessing the causal effect of TB-BMD on circulating SHBG level. Figure S10. Plots of “leave-one-out” analyses for the reverse MR analyses assessing the causal effect of TB-BMD on circulating SHBG level. [file 12891_2025_8956_MOESM3_ESM.zip › Additional File 3 Figure S6.tif]

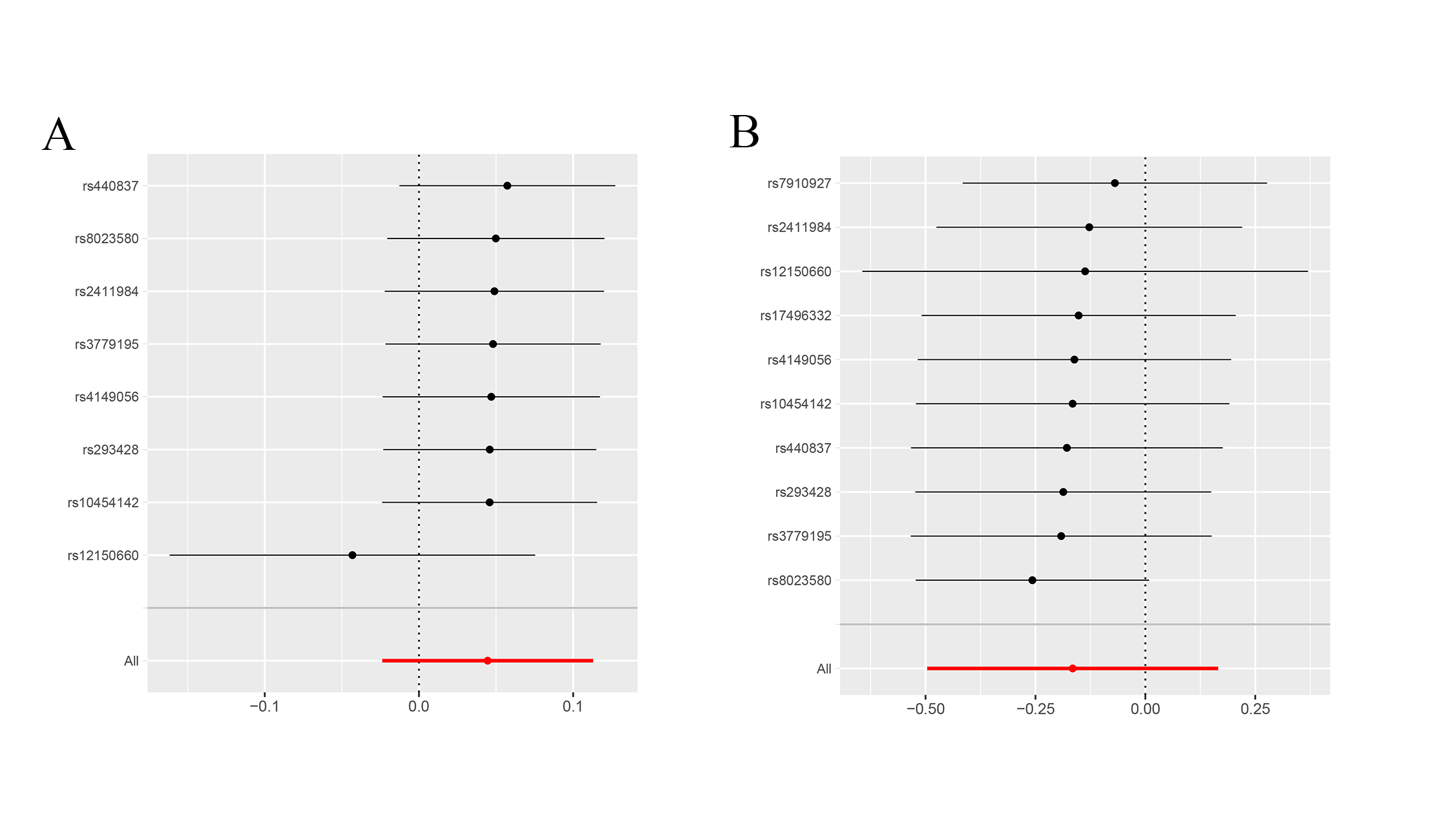

Supplement: Supplementary file 3 — Additional file 3. Figure S1. Funnel plots for MR analyses assessing the causal effect of circulating SHBG on BMDs using the first set of SNPs (A) FN-BMD (B) LS-BMD (C) FA-BMD (D) TB-BMD. Figure S2. Funnel plots for MR analyses assessing the causal effect of circulating SHBG on BMDs using the second set of SNPs (A) FN-BMD (B) LS-BMD (C) FA-BMD (D) TB-BMD. Figure S3. Plots of “leave-one-out” analyses for MR analyses assessing the causal effect of circulating SHBG on BMDs using the first set of SNPs (A) FN-BMD (B) LS-BMD (C) FA-BMD (D) TB-BMD. Figure S4. Plots of “leave-one-out” analyses for MR analyses assessing the causal effect of circulating SHBG on BMDs using the second set of SNPs (A) FN-BMD (B) LS-BMD (C) FA-BMD (D) TB-BMD. Figure S5. Funnel plots for MR analyses assessing the causal effect of circulating SHBG on BMI and T2DM using the first set of SNPs (A) BMI (B) T2DM. Figure S6. Funnel plots for MR analyses assessing the causal effect of circulating SHBG on BMI and T2DM using the second set of SNPs (A) BMI (B) T2DM. Figure S7. Plots of “leave-one-out” analyses for MR analyses assessing the causal effect of circulating SHBG on BMI and T2DM using the first set of SNPs (A) BMI (B) T2DM. Figure S8. Plots of “leave-one-out” analyses for MR analyses assessing the causal effect of circulating SHBG on BMI and T2DM using the second set of SNPs (A) BMI (B) T2DM. Figure S9. Funnel plots for the reverse MR analyses assessing the causal effect of TB-BMD on circulating SHBG level. Figure S10. Plots of “leave-one-out” analyses for the reverse MR analyses assessing the causal effect of TB-BMD on circulating SHBG level. [file 12891_2025_8956_MOESM3_ESM.zip › Additional File 3 Figure S7.tif]

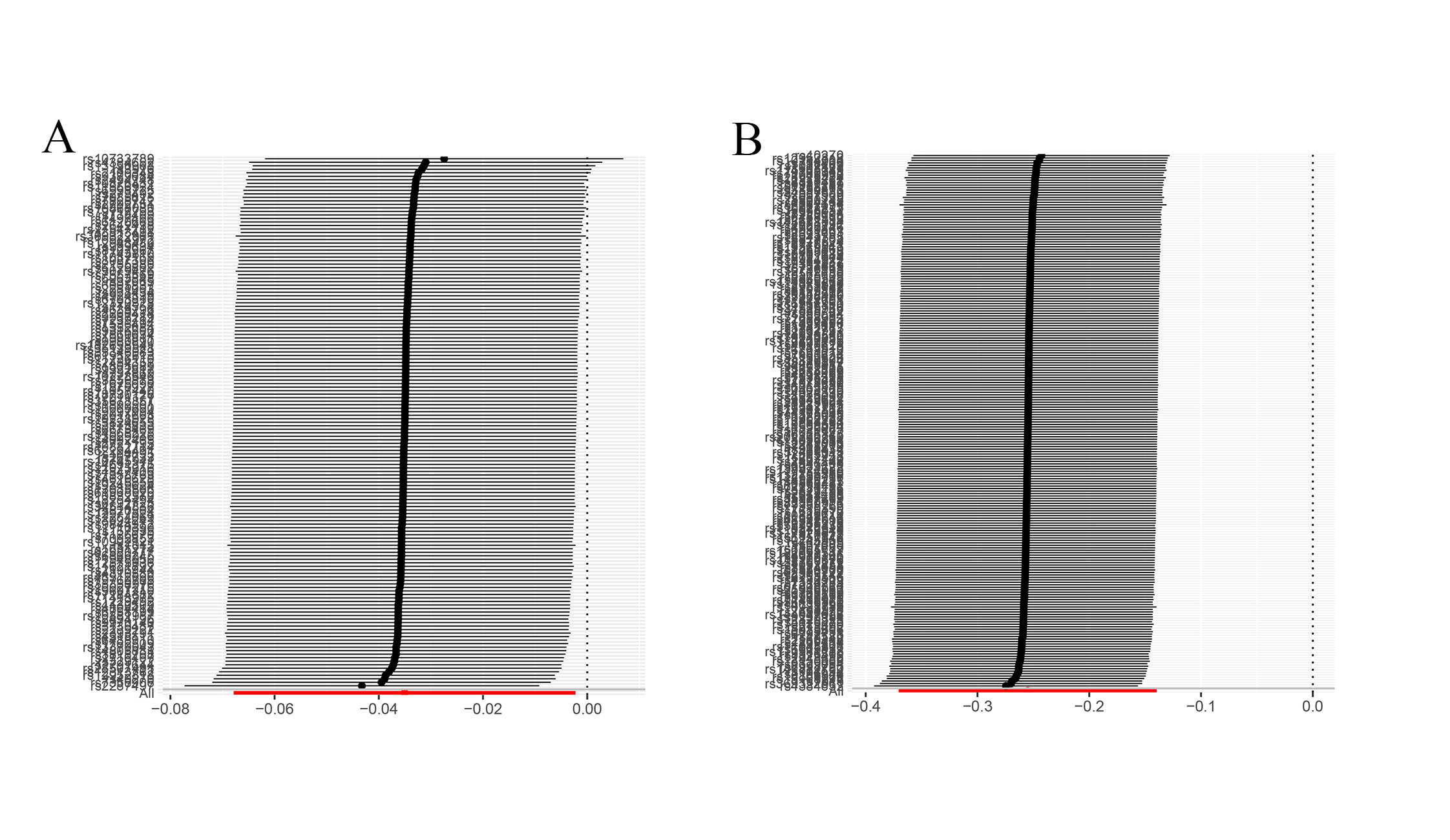

Supplement: Supplementary file 3 — Additional file 3. Figure S1. Funnel plots for MR analyses assessing the causal effect of circulating SHBG on BMDs using the first set of SNPs (A) FN-BMD (B) LS-BMD (C) FA-BMD (D) TB-BMD. Figure S2. Funnel plots for MR analyses assessing the causal effect of circulating SHBG on BMDs using the second set of SNPs (A) FN-BMD (B) LS-BMD (C) FA-BMD (D) TB-BMD. Figure S3. Plots of “leave-one-out” analyses for MR analyses assessing the causal effect of circulating SHBG on BMDs using the first set of SNPs (A) FN-BMD (B) LS-BMD (C) FA-BMD (D) TB-BMD. Figure S4. Plots of “leave-one-out” analyses for MR analyses assessing the causal effect of circulating SHBG on BMDs using the second set of SNPs (A) FN-BMD (B) LS-BMD (C) FA-BMD (D) TB-BMD. Figure S5. Funnel plots for MR analyses assessing the causal effect of circulating SHBG on BMI and T2DM using the first set of SNPs (A) BMI (B) T2DM. Figure S6. Funnel plots for MR analyses assessing the causal effect of circulating SHBG on BMI and T2DM using the second set of SNPs (A) BMI (B) T2DM. Figure S7. Plots of “leave-one-out” analyses for MR analyses assessing the causal effect of circulating SHBG on BMI and T2DM using the first set of SNPs (A) BMI (B) T2DM. Figure S8. Plots of “leave-one-out” analyses for MR analyses assessing the causal effect of circulating SHBG on BMI and T2DM using the second set of SNPs (A) BMI (B) T2DM. Figure S9. Funnel plots for the reverse MR analyses assessing the causal effect of TB-BMD on circulating SHBG level. Figure S10. Plots of “leave-one-out” analyses for the reverse MR analyses assessing the causal effect of TB-BMD on circulating SHBG level. [file 12891_2025_8956_MOESM3_ESM.zip › Additional File 3 Figure S8.tif]

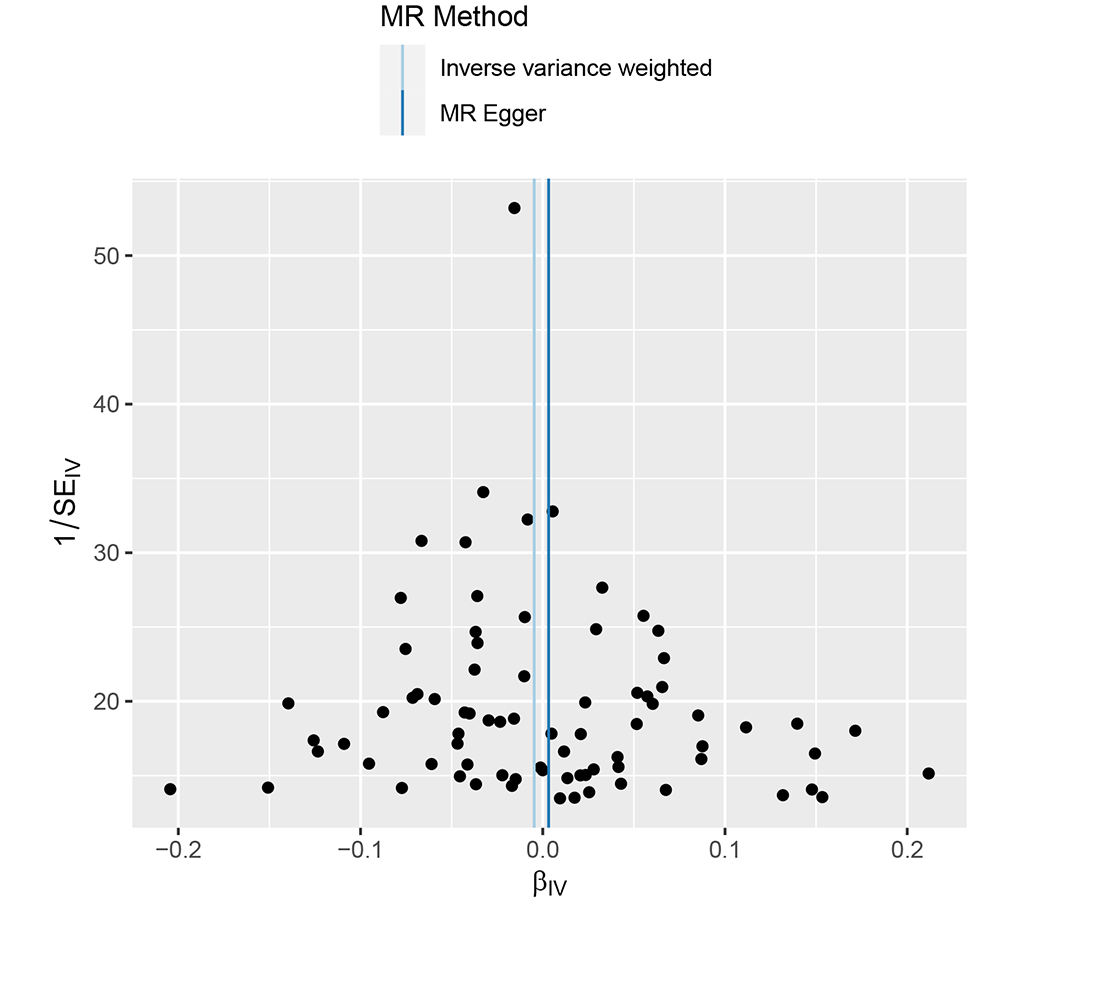

Supplement: Supplementary file 3 — Additional file 3. Figure S1. Funnel plots for MR analyses assessing the causal effect of circulating SHBG on BMDs using the first set of SNPs (A) FN-BMD (B) LS-BMD (C) FA-BMD (D) TB-BMD. Figure S2. Funnel plots for MR analyses assessing the causal effect of circulating SHBG on BMDs using the second set of SNPs (A) FN-BMD (B) LS-BMD (C) FA-BMD (D) TB-BMD. Figure S3. Plots of “leave-one-out” analyses for MR analyses assessing the causal effect of circulating SHBG on BMDs using the first set of SNPs (A) FN-BMD (B) LS-BMD (C) FA-BMD (D) TB-BMD. Figure S4. Plots of “leave-one-out” analyses for MR analyses assessing the causal effect of circulating SHBG on BMDs using the second set of SNPs (A) FN-BMD (B) LS-BMD (C) FA-BMD (D) TB-BMD. Figure S5. Funnel plots for MR analyses assessing the causal effect of circulating SHBG on BMI and T2DM using the first set of SNPs (A) BMI (B) T2DM. Figure S6. Funnel plots for MR analyses assessing the causal effect of circulating SHBG on BMI and T2DM using the second set of SNPs (A) BMI (B) T2DM. Figure S7. Plots of “leave-one-out” analyses for MR analyses assessing the causal effect of circulating SHBG on BMI and T2DM using the first set of SNPs (A) BMI (B) T2DM. Figure S8. Plots of “leave-one-out” analyses for MR analyses assessing the causal effect of circulating SHBG on BMI and T2DM using the second set of SNPs (A) BMI (B) T2DM. Figure S9. Funnel plots for the reverse MR analyses assessing the causal effect of TB-BMD on circulating SHBG level. Figure S10. Plots of “leave-one-out” analyses for the reverse MR analyses assessing the causal effect of TB-BMD on circulating SHBG level. [file 12891_2025_8956_MOESM3_ESM.zip › Additional File 3 Figure S9.tif]
